# A narrative systematic review of school-based screening tools to identify Autism Spectrum Disorders

**DOI:** 10.1192/j.eurpsy.2026.10565

**Published:** 2026-07-31

**Authors:** L. J. McCann, R. Bakhti, N. Fonseka, D. Nicholls, D. Hargreaves, F. Amati, A. Lazzarino, R. Mitra, K. Narayan, A. Weston, S. Gnani

**Affiliations:** 1 Department of Twin Research & Genetic Epidemiology London, King’s College London, UK; 2 Department of Brain Sciences, Faculty of Medicine, Imperial College London, London; 3 School of Medicine, Faculty of Medicine and Health Sciences, Keele University, Staffordshire; 4 School of Public Health, Faculty of Medicine, Imperial College London; 5 Central and Northwest London NHS Foundation Trust; 6 The Giaroli Centre; 7 Institute of Psychology Psychiatry and Neuroscience, King’s College London; 8 Listen to Act, London, United Kingdom

## Abstract

**Introduction:**

Autism Spectrum Disorder (ASD) is a common neurodevelopmental condition characterized by challenges in social interaction, communication, and repetitive behaviours. Early identification through school-based screening for ASD has the potential to enhance educational and health outcomes for affected children.

**Objectives:**

This review examines school-based screening tools for identifying children and young people with ASD, and assess the differences and challenges in effectiveness across various socio-demographic groups.

**Methods:**

We conducted a narrative systematic review and searched EMBASE, MEDLINE, PsychINFO, Cochrane, and Scopus electronic databases (2011-2024) in October 2024 to identify studies on school-based ASD screening for children aged 4 to 16 years. Two independent researchers screened the papers and extracted the data. Data extracted included country, age, gender, socio-economic status, ethnicity, type of screening tool, informant details, and outcomes (prevalence of screen positives, sensitivity, and specificity).

**Results:**

Of 7,311 eligible articles, 14 studies were included in this review. Eight different ASD school-based screening tools were identified. Study populations ranged from 103 to 16,556 participants, with varying sensitivity and specificity depending on the population age group, setting, and ASD prevalence. The prevalence of screening positive for ASD ranged from 0.7% to 8.5%. Most studies were conducted in European countries (n=6) followed by Western Pacific (n=4), Americas (n=3) and Eastern Mediterranean (n=1) countries. No studies evaluated outcomes based on ethnicity or socioeconomic status.

**Conclusions:**

There is a broad range of school-based ASD screening tools available globally, but their use is inconsistent and lacks robust validation. Evidence on diagnostic accuracy remains limited, and the dearth of data on ethnicity and socio-economic status risks exacerbating health inequalities. Consensus on tool selection and validation, alongside collaboration between education systems, researchers, and policymakers, is needed to ensure ASD screening approaches are equitable and effective.

**Disclosure of Interest:**

None Declared

